# Individual differences in human fear generalization—pattern identification and implications for anxiety disorders

**DOI:** 10.1038/s41398-019-0646-8

**Published:** 2019-11-18

**Authors:** Y. Stegmann, M. A. Schiele, D. Schümann, T. B. Lonsdorf, P. Zwanzger, M. Romanos, A. Reif, K. Domschke, J. Deckert, M. Gamer, P. Pauli

**Affiliations:** 1 Department of Psychology, University of Würzburg, Würzburg, Germany; 2 Center for Mental Health, University of Würzburg, Würzburg, Germany; 3 Department of Psychiatry and Psychotherapy, Medical Center - University of Freiburg, Faculty of Medicine, University of Freiburg, Freiburg, Germany; 4 Department of Systems Neuroscience, University Medical Center Hamburg-Eppendorf, Hamburg, Germany; 5 kbo-Inn-Salzach-Hospital, Wasserburg, Germany; 6 Department of Psychiatry, University of Münster, Münster, Germany; 7 Department of Psychiatry, Ludwig-Maximilian-University of Munich, München, Germany; 8 Department of Child and Adolescent Psychiatry, Psychosomatics and Psychotherapy, Center for Mental Health, University Hospital Würzburg, Würzburg, Germany; 9 Department of Psychiatry, Psychosomatic Medicine and Psychotherapy, University Hospital Frankfurt, Frankfurt am Main, Germany; 10 Center for NeuroModulation, Faculty of Medicine, University of Freiburg, Freiburg, Germany; 11 Department of Psychiatry, Psychosomatics and Psychotherapy, Center for Mental Health, University Hospital Würzburg, Würzburg, Germany

**Keywords:** Human behaviour, Psychiatric disorders

## Abstract

Previous research indicates that anxiety disorders are characterized by an overgeneralization of conditioned fear as compared with healthy participants. Therefore, fear generalization is considered a key mechanism for the development of anxiety disorders. However, systematic investigations on the variance in fear generalization are lacking. Therefore, the current study aims at identifying distinctive phenotypes of fear generalization among healthy participants. To this end, 1175 participants completed a differential fear conditioning phase followed by a generalization test. To identify patterns of fear generalization, we used a k-means clustering algorithm based on individual arousal generalization gradients. Subsequently, we examined the reliability and validity of the clusters and phenotypical differences between subgroups on the basis of psychometric data and markers of fear expression. Cluster analysis reliably revealed five clusters that systematically differed in mean responses, differentiation between conditioned threat and safety, and linearity of the generalization gradients, though mean response levels accounted for most variance. Remarkably, the patterns of mean responses were already evident during fear acquisition and corresponded most closely to psychometric measures of anxiety traits. The identified clusters reliably described subgroups of healthy individuals with distinct response characteristics in a fear generalization test. Following a dimensional view of psychopathology, these clusters likely delineate risk factors for anxiety disorders. As crucial group characteristics were already evident during fear acquisition, our results emphasize the importance of average fear responses and differentiation between conditioned threat and safety as risk factors for anxiety disorders.

## Introduction

Adaptive responses triggered by fear and anxiety are essential for survival. By contrast, non-adaptive and irrational fears are key features of anxiety disorders, which constitute the most prevalent class of psychiatric disorders and pose a heavy burden on the public healthcare systems^1^. Their impact on society and on the quality of life of affected individuals has largely been underestimated in the past^2^. These facts thus highlight the importance of investigating mechanisms that are responsible for the development and maintenance of anxiety disorders with the aim to identify and improve prevention and treatment strategies. An important step toward this aim is the identification of subgroups based on biopsychological characteristics that may help to identify risk and protective factors for anxiety disorders and call for different treatment strategies as discussed in the context of personalized medicine^3^.

Fear conditioning is the prevailing model to elucidate the processes underlying the development of phobias and other anxiety disorders^4^. During the acquisition phase of differential fear conditioning paradigms, one stimulus (CS +) is repeatedly paired with an aversive stimulus (unconditioned stimulus, US), while another stimulus (CS−) is never paired with the US. As a result, the presentation of the CS + elicits fear responses by gaining predictive value for the aversive US^5^. In these models, fear generalization describes the transfer of conditioned fear from a threatening stimulus, e.g., the CS +, to stimuli that share some similarity with the threatening stimulus (called generalization stimuli, GS) but have never been associated with the US^6,7^. Such generalization processes are discussed as crucial for the transformation from adaptive to pathological fear^8^.

Fear generalization has been demonstrated for GSs that share perceptual characteristics with the CS +, e.g., with respect to size^7^, color^9^, or orientation^10^, but it was also observed for similarity on a categorical dimension^11^ or for contexts^12^. The degree of generalization can be characterized by a generalization gradient^13^, which normally gradually declines as the similarity to the CS + decreases^7^. Steeper gradients and quadratic as compared with linear trends indicate less fear generalization^14^. Using this approach, several studies reported that patients with different kinds of anxiety and stress-related disorders (e.g., PTSD^13^, panic disorder^15^, and GAD^14^) tend to show more linear gradients compared to healthy controls. However, such overgeneralization could not be demonstrated consistently^16^ and not for all anxiety disorders, e.g., social anxiety disorder^17^, questioning the significance of overgeneralization as a pathogenic marker for anxiety disorders. In addition, a meta-analysis allowing to estimate the effect size of studies examining overgeneralization in patient groups is missing. As a consequence, researchers suggested novel analytic approaches to the study of fear generalization^18^ and started to employ modern cluster-led mixed-model methods to examine whether fear generalization merely reflects a failure to perceptually discriminate between stimuli^19,20^. Another reason for the lack of convergent findings may be due to the fact that a majority of the studies so far did not thoroughly discuss whether differences in generalization may be related to more basic characteristics such as mean fear response levels or efficacy of conditioning. Although there is some evidence, that anxiety patient and control groups differ in fear generalization but not in CS-differentiation during acquisition^13–15^, some previous studies also reported group differences in measures unrelated to fear generalization gradients (e.g., heightened startle responses of anxiety disorder patients in the intertrial interval^15^, or higher risk ratings to the CS + for PTSD patients compared to controls^13^. Because of its complexity it seems appropriate to consider fear generalization as a multi-dimensional mechanism. Unraveling these dimensions may be crucial to appraise the importance of generalization as an independent risk factor for anxiety disorders.

Whereas previous research mainly focused on clear distinctions between groups (e.g., patients vs. healthy controls), it has been acknowledged that psychopathology might be better conceptualized as a continuum (cf., Research Domain Criteria, RDoC^21,22^). Following this approach, we assume that individual differences in fear generalization also exist in a healthy population. However, research on differences in fear generalization among healthy individuals is scarce, although the need to identify meaningful factors contributing to the substantial individual variability in fear generalization has been acknowledged^8^.

Therefore, in the current study, we used cluster analysis on a large sample of healthy participants to identify homogeneous subgroups characterized by distinct patterns of individual fear generalization gradients. In contrast to the regularly employed analytical approaches, the cluster analysis does not need a priori assumptions about the shape of individual generalization profiles and thus is perfectly suited to reveal systematic covariation of multiple dimensions contributing to interindividual variance in fear generalization. We then examined cluster stability by evaluating different measures of generalization and cluster validity by examining phenotypical differences between the obtained subgroups regarding fear conditioning and psychometric measures of psychopathology. Since this is the first study realizing this approach, we made no a priori assumptions about the number of clusters but expected that subgroups characterized by fear overgeneralization show enhanced fear and anxiety traits.

## Materials and methods

### Sample

In total, 1 175 healthy participants were recruited within a Collaborative Research Center on fear and anxiety (SFB-TRR58) at the Universities of Würzburg, Münster, and Hamburg, Germany. All participants took part in the initial recruitment phase (2013–2016) and completed a differential fear conditioning phase followed by a generalization test. They also were characterized phenotypically with psychometric questionnaires (see below). Exclusion criteria included left-handedness, non-Caucasian descent, intake of psychoactive medication, excessive consumption of alcohol, nicotine, and caffeine, consumption of illegal drugs, severe medical diseases, or being pregnant^23,24^. The absence of current and/or lifetime diagnosis of DSM-IV mental Axis-I disorders was assessed by the German version of the Mini International Psychiatric Interview^25^. All volunteers gave written informed consent and were paid 50 Euros. The study was approved by the ethic committees of the involved Universities. All procedures were in agreement with the Declaration of Helsinki (Version 2008).

### Psychometric assessment

Prior to the experiment, participants completed several questionnaires (see Table 1) including the trait version of the State-Trait Anxiety Inventory^26^, the Anxiety Sensitivity Index 3^27,28^, the Agoraphobic Cognitions Questionnaire^29,30^, the Liebowitz Social Anxiety Scale^31,32^, and the Social Phobia and Anxiety Inventory^33^ to assess general and specific symptoms of anxiety. Symptoms of depression were measured by the short version of the Center for Epidemiological Studies-Depression Scale^34^. The Childhood Trauma Questionnaire^35,36^ was used for a retrospective assessment of childhood maltreatment. Additionally, the General Self-Efficacy Scale^37^ and the Behavioral Inhibition Scale^38,39^ were included, since these traits were suggested to correlate with symptoms of anxiety^40,41^.

**Table 1:** Summary of the sample characteristics.

	Overall sample (*n* = 1 175)				Female (*n* = 686)				Male (*n* = 489)			
	M	SD	Min	Max	M	SD	Min	Max	M	SD	Min	Max
Age (years)	25.7	5.9	18	50	25.2	6.2	18	50	25.8	5.2	18	49
STAI-T	34.6	8.2	20	67	35.2	8.2	20	67	33.7	8.0	20	66
ASI-3	12.1	8.3	0	48	12.0	8.2	0	45	12.2	8.5	0	48
ACQ	1.3	0.2	1.0	2.7	1.4	0.2	1.0	2.5	1.3	0.2	1.0	2.7
PSWQ	40.4	9.8	17	73	42.3	10.0	19	73	37.8	9.1	17	67
SPAI	33.2	17.0	0	103.7	34.5	16.9	0	103.7	31.2	16.9	0	99.2
LSAS	21.4	15.3	0	90	21.9	15.1	0	90	20.6	15.5	0	84
CES-D	6.9	5.7	0	38	6.8	5.9	0	38	6.9	5.4	0	28
BIS	2.8	0.5	1.1	4	2.9	0.5	1.1	4	2.6	0.5	1.1	4
GSE	30.0	3.7	15	40	29.6	3.7	17	40	30.5	3.7	15	40
CTQ	32.1	8.1	25	97	32.9	8.2	25	74	32.4	7.9	25	97

*M* mean, *SD* standard deviation, *Min* minimum, *Max* maximum, *STAI-T* State-Trait Anxiety Inventory – Trait, ASI-3 Anxiety Sensitivity Index 3, *ACQ* Agoraphobic Cognition Questionnaire, *SPAI* Social Phobia and Anxiety Inventory, *LSAS* Liebowitz Social Anxiety Scale, *CES-D* Center for Epidemiological Studies-Depression Scale, *BIS* Behavioral Inhibition Scale, *GSE* General Self-Efficacy Scale, *CTQ* Childhood Trauma Questionnaire

### Fear acquisition and generalization tasks

Participants completed a differential fear conditioning paradigm as developed by Lau et al.^42^, in which two female faces with neutral expression served as conditioned stimuli (CS). The paradigm consisted of three phases: During pre-acquisition each of the two faces was individually presented four times for a duration of 6 s each (eight trials). In the acquisition phase, participants saw 12 times the CS + and CS− (24 trials). Ten CS + presentations (83.3% reinforcement rate) were followed by the US, consisting of a fearful facial expression of the same person as the CS + with a simultaneous presentation of a 95 dB loud female scream for a duration of 1.5 s. During generalization test, four generalization stimuli (GS) were presented in addition to the CS. Generalization stimuli were morphs of the original CS faces in 20% steps. Each stimulus was presented 12 times (72 trials). Half of CS + presentations were paired with the US to prevent rapid extinction. Participants were not instructed about the CS-US contingencies and the assignment of faces to CS + and CS− was randomized across participants. After half of the total acquisition and generalization trials and at the end of each phase, participants were asked to rate the faces regarding valence, arousal (both 9-point Likert-scales; from 1 = very unpleasant/very calm to 9 = very pleasant/very arousing) and US-contingency (11-point Likert-scale; from 0 to 100% in 10% increments). Please note that valence ratings were inverted for subsequent analyses to increase comparability. We additionally recorded skin conductance responses (SCR) as a psychophysiological marker of fear expression.

### Physiological data processing

Using a constant-voltage system (0.5 V), SCR were recorded from the thenar and hypothenar eminences of the left hand with Ag/AgCl electrodes. Signals were amplified and recorded using a V-Amp-16 and Vision Recorder software (Brainproducts, Gilching, Germany) at a sampling rate of 1 000 Hz. Offline data processing within the Vision Analyzer 2 software included filtering with a high cutoff filter of 1 Hz and a notch filter of 50 Hz. SCR amplitude was defined as the base-to-peak difference in μS between response onset (900–4000 ms after stimulus onset) and peak (2000–6000 ms after stimulus onset)^43^. A minimum response criterion of 0.02 μS was applied, with lower responses scored as 0. To compensate for skewed distributions, we employed square-root transformations.

### Cluster analysis

The purpose of this cluster analysis was to identify relatively homogeneous subgroups within the total sample based on distinct patterns in individual fear generalization gradients. We relied on individual arousal ratings during the generalization test, since they are well suited to track affective responses underlying fear expression^5^ and had the highest mutual correlation to valence and US-contingency ratings. For the classification of participants, we used a k-means clustering algorithm^44^, implemented in the R software environment (version 3.5.0, https://www.r-project.org). This algorithm allows for dividing the whole group of participants, each characterized by a set of variables, into a number of k clusters. Participants are iteratively relocated to specific clusters until within-cluster sum of squares converges to a minimum. Variables entered in the cluster analysis were the individual arousal ratings to the conditioned (CS + and CS−) and generalization stimuli (GS1–GS4), after averaging over both rating trials during generalization. All six variables were z-standardized across participants prior entering the analysis. The sum of squares that is minimized during the clustering algorithm reflects the sum of squared Euclidean distances between participants’ arousal ratings (CS+ to CS−) and cluster centroids. The number of clusters was determined by inspecting the within-cluster sum of squares scree-plot, by analyzing 25 indices for objectively determining the relevant numbers of clusters, as provided by the R package ‘NbClust’^45^, and by interpretability of the emerging cluster solutions.

The resulting clusters were characterized by comparing the individually averaged generalization gradients between subgroups. To this end, we calculated three different indices for each subject: The mean score of the responses to all six conditioned and generalization stimuli (mean responses); the difference between CS+ and CS− responses (CS-differentiation); and the linear deviation score (LDS)^13,46^ as an index of linearity of the generalization gradient, calculated as the difference of the mean response to the conditioned stimuli minus the mean response to the generalization stimuli [(CS+ + CS−)/2 – (GS1 + GS2 + GS3 + GS4)/4].

To determine the stability of the clusters, the study sample was randomly divided into two roughly equal-sized subsamples. If clusters are stable, cluster analyses in both groups should reveal similar cluster structures^47^. The cluster solution was also validated by comparing subgroups on valence and US-contingency ratings as well as skin conductance responses during acquisition and generalization.

Differences between clusters in indices of fear responding and psychometric characteristics were analyzed by separate univariate ANOVAs with the between-factor cluster assignment. Subgroup differences were post hoc analyzed by computing Scheffé-tests, which account for unequal sample sizes and are the most conservative post hoc tests and thus appropriate for the explorative nature of these analyses^48,49^. The significance level was set to alpha = 0.05.

## Results

### Number of clusters

Cluster analyses using arousal ratings led to a within-cluster sum of squares scree-plot suggesting either a four, five or six cluster solution (see Supplemental Fig. S1). We also calculated 25 different indices for objectively determining the optimal number of clusters (for a summary on these indices, see ref. ^45^). Seven indices proposed five as the optimal number of clusters, whereas only 1 and even 0 among the 25 indices suggested a four or a six cluster solution, respectively. Therefore, five clusters were finally selected for further analyses, and this decision was retrospectively supported by distinctive cluster characteristics (see below).

### Cluster characteristics

**Fig. 1:**
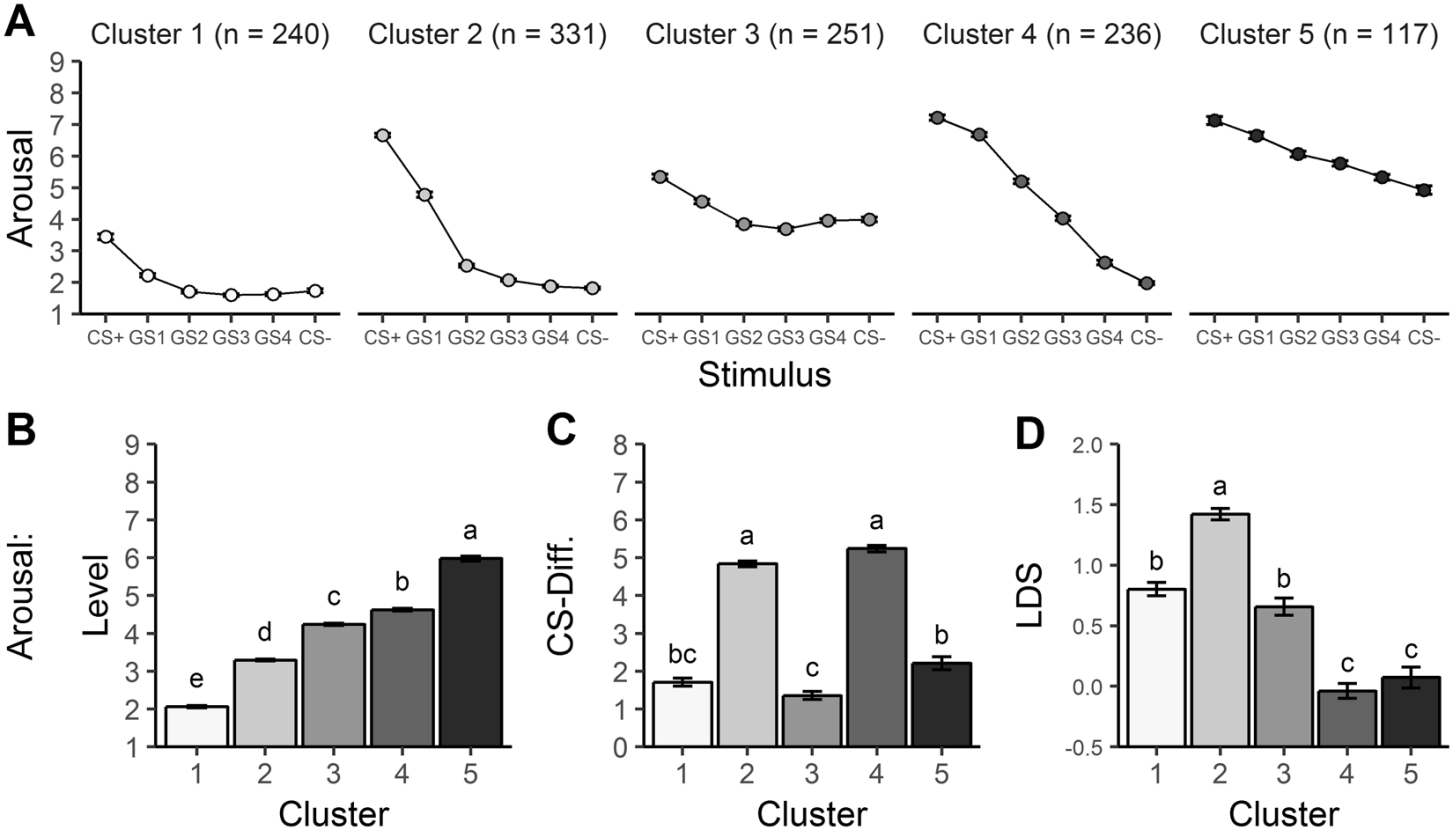
Cluster characteristics for arousal ratings during the generalization test. Arousal generalization gradients for each cluster (**a**) and corresponding gradient parameters (**b**–**d**). Mean arousal responses (**b**) increased from clusters 1–5; CS-differentiation (**c**) was better in clusters 2 and 4 than clusters 1, 3, and 5; linear deviation scores (LDS; **d**) indicate more linear gradients in clusters 4 and 5 than clusters 1, 2, and 3. Clusters with the same letters do not differ on a Scheffé-corrected alpha level of 0.05. Error bars represent standard errors of the mean.

The five clusters differed significantly in mean arousal level, *F*(4,1170) = 1450.50, *p* *<* 0.001, $\eta _p^2$ = 0.832, CS-differentiation, *F*(4,1170) = 348.06, *p* *<* 0.001, $\eta _p^2$ = 0.543, and linear deviation score (LDS), *F*(4,1170) = 96.84, *p* *<* 0.001, $\eta _p^2$ = 0.249 (see Supplemental Table S1). Ranking the clusters according to their respective mean arousal levels yielded Fig. 1 (which also depicts post hoc tests). Consequently, cluster 1 (*n* = 240) is characterized by the lowest arousal level, which incrementally increases for cluster 2 (*n* = 331), cluster 3 (*n* = 251), cluster 4 (*n* = 236), and finally cluster 5 (*n* = 117) marked by the highest mean arousal level. Clusters 2 and 4 showed stronger CS-differentiation than the remaining clusters. Finally, linearity of generalization (LDS) is strongest in clusters 4 and 5, which differed from clusters 1, 2, and 3, while cluster 1 and 3 also showed more linear gradients compared with cluster 2. These differences in cluster characteristics suggest that generalization gradients are adequately described not only by their deviation from linearity, but also by mean response level and CS-differentiation. Notably, we found small but significant correlations in the full sample between mean arousal levels and CS-differentiation, *r*(1173) = 0.12, *p* *<* 0.001, mean arousal levels and LDS, *r*(1173) = −0.29, *p* *<* 0.001, and CS-differentiation and LDS, *r*(1173) = 0.19, *p* *<* 0.001.

### Cluster stability

Dividing the whole sample into two roughly equal-sized halves and then repeating the cluster analysis yielded the same cluster structure and similar relative cluster sizes in both subsamples (see Supplemental Fig. S2).

To further test the cluster stability, the identified clusters were compared regarding the three generalization gradients’ parameters (mean response, CS-differentiation, LDS) derived from the other measures of generalization. For both valence and US-contingency ratings (see Supplement Figs S3–S4 and Supplemental Table S1), significant differences were observed for all three parameters: valence [level: *F*(4,1170) = 136.99, *p* *<* 0.001, $\eta _p^2$ = 0.319, CS-differentiation: *F*(4,1170) = 152.16, *p* *<* 0.001, $\eta _p^2$ = 0.342, LDS: *F*(4,1170) = 52.32, *p* *<* 0.001, $\eta _p^2$ = 0.152], US-contingency [level: *F*(4,1170) = 49.88, *p* *<* 0.001, $\eta _p^2$ = 0.146, CS-differentiation: *F*(4,1170) = 37.88, *p* *<* 0.001, $\eta _p^2$ = 0.115, LDS: *F*(4,1170) = 22.09, *p* *<* 0.001, $\eta _p^2$ = 0.070]. For both measures, the means increased from clusters 1 to 5 (without significant differences between cluster 1 and 2 and between clusters 3 and 4) and the CS-differentiation was greater for cluster 2 and cluster 4 than the remaining clusters. In addition, LDS was for both valence and US-contingency stronger in cluster 5 and cluster 4 compared with the other clusters.

**Fig. 2:**
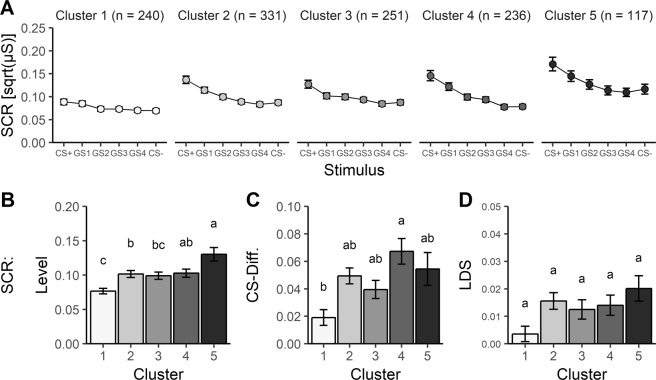
Cluster characteristics for skin conductance responses during the generalization test. Skin conductance response (SCR) generalization gradients for each cluster (**a**) and corresponding gradient parameters (**b**–**d**). Mean SCRs differed between cluster 1 and 5 with clusters 2–4 in between (**b**); CS-differentiation was better in cluster 1 than in cluster 4 (**c**); no significant differences were found for linear deviation scores (LDS) (**d**). Clusters with the same letters do not differ on a Scheffé-corrected alpha level of 0.05. Error bars represent standard errors of the mean.

Skin conductance responses grossly reflected this pattern of generalization too as clusters differed in mean SCRs, *F*(4,1170) = 8.12, *p* *<* 0.001, $\eta _p^2$ = 0.027, CS-differentiation, *F*(4,1170) = 5.86, *p* *<* 0.001, $\eta _p^2$ = 0.019, and LDS, *F*(4,1170) = 2.64, *p* *=* 0.032, $\eta _p^2$ = .009 (see Fig. 2 and Supplemental Table S2). However, the follow-up tests—although confirming the pattern grossly—revealed less differences between clusters: Mean skin conductance responses were higher in cluster 5 compared with clusters 1-3 as well as in clusters 2 and 4 compared to cluster 1, CS-differentiation only differed between cluster 4 and cluster 1, and post hoc tests revealed no significant LDS differences.

In sum, we revealed convincing cluster stability. Clusters could be replicated in subsamples and showed very similar characteristics for arousal, valence and US-contingency ratings, and largely similar characteristics for skin conductance responses. Tentatively, cluster 5 may be considered a risk cluster as it is characterized by strongest fear generalization, but also by strongest mean fear responses and low CS-differentiation.

### Cluster validity

**Fig. 3:**
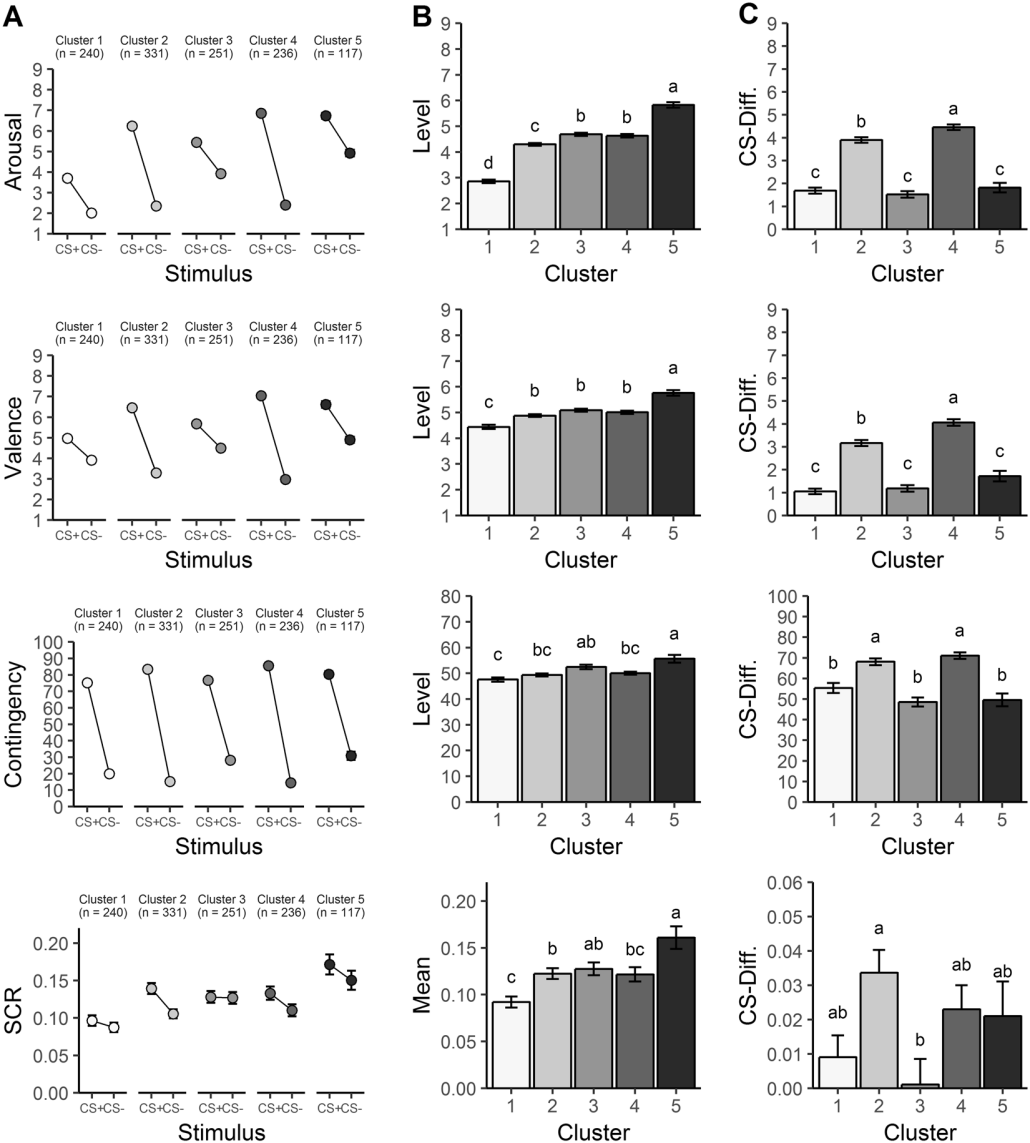
Cluster characteristics during the acquisition phase. Ratings and skin conductance responses (SCR) for CS+ and CS− during acquisition (**a**) and the extracted characteristics reflected in mean responses (**b**) and CS-differentiation (**c**). The clusters’ response characteristics identified during the generalization phase (see Fig. 1) were already evident during acquisition (see text for detailed explanation). Clusters with the same letters do not differ on a Scheffé-corrected alpha level of 0.05. Error bars represent standard errors of the mean.

To test for potential predictor variables of cluster membership, we first compared indices of fear acquisition between clusters, i.e., we compared clusters regarding mean responses and CS-differentiation during acquisition. Importantly, the characteristic cluster patterns for arousal, valence and US-contingency ratings were already established after acquisition, all *p*’s < 0.001, (see Fig. 3 and Supplemental Table S2). Post hoc tests revealed for arousal, valence and US-contingency ratings higher mean ratings for cluster 5 than cluster 1 with clusters 2–4 in between, and stronger CS-differentiation for clusters 2 and 4 than clusters 1, 3, and 5. Mean skin conductance responses also yielded higher responses for cluster 5 than cluster 1 with clusters 2–4 in between, but CS-differentiation only differed between clusters 2 and 3.

Second, we compared clusters regarding psychometric data and found significant cluster differences for most questionnaires (see Table 2). Follow-up tests specified that these effects were due to differences between cluster 5 and cluster 1, with cluster 5 having higher scores in trait-anxiety (STAI-T), anxiety sensitivity (ASI-3), agoraphobic cognitions (ACQ), social anxiety (LSAS and SPAI), and behavioral inhibition (BIS), and lower scores in general self-efficacy (GSE). On every questionnaire, clusters 2, 3, and 4 scored between cluster 1 and 5 in ascending order (descending for general self-efficacy), all *p*s < 0.001. However, effect sizes were low with all $\eta _p^2$s < 0.027. No questionnaire data differentiated significantly between clusters 2, 3, and 4, and no statistically significant differences between clusters were found for general depression scores (CES-D) and the childhood trauma questionnaire (CTQ).

**Table 2:** Cluster comparisons on questionnaire data.

	Cluster 1		Cluster 2		Cluster 3		Cluster 4		Cluster 5		Statistics			Cluster:
	(*n* = 240)		(*n* = 331)		(*n* = 251)		(*n* = 236)		(*n* = 117)					1 - 2 - 3 - 4 - 5
	*n*	%	*n*	%	*n*	%	*n*	%	*n*	%	*χ²(4)*	*p*		
Female	133	55.4	196	59.2	124	49.4	159	67.4	74	63.2	18.28	<0.001		
	M	SD	M	SD	M	SD	M	SD	M	SD	*F*(4,1170)	*p*	$\eta _p^2$	Scheffé^a^
Age (years)	26.1	6.4	25.7	5.9	25.4	5.6	25.5	5.8	25.4	5.1	0.66	0.623	0.002	n.s.
STAI-T	32.7	7.9	34.0	7.9	35.4	8.2	35.1	8.2	37.4	8.5	8.09	<0.001	0.027	c - bc - ab - ab - a
ASI-3	10.0	7.5	11.8	8.1	12.8	9.1	12.5	7.7	14.9	8.9	7.99	<0.001	0.027	c - bc - ab - ab - a
ACQ	1.3	0.2	1.3	0.2	1.4	0.3	1.4	0.2	1.4	0.2	5.94	<0.001	0.02	b - ab - ab - a - a
SPAI	28.9	16.3	32.6	15.8	33.3	17.1	35.2	17.9	38.7	17.0	8.08	<0.001	0.027	c - bc - abc - ab - a
LSAS	17.7	13.2	20.6	14.7	22.2	15.7	23.1	16.0	26.4	16.7	7.93	<0.001	0.026	c - bc - ab - ab - a
CES-D	6.2	5.6	6.6	5.5	7.0	5.6	7.3	6.0	7.8	6.0	2.29	0.058	0.008	n.s.
BIS	2.6	0.5	2.8	0.5	2.7	0.5	2.8	0.5	2.9	0.5	6.83	<0.001	0.023	c - ab - bc - ab - a
GSE	30.8	3.6	30.1	3.6	29.7	3.9	29.6	3.8	29.5	3.4	4.77	<0.001	0.016	a - ab - b - b - b
CTQ	31.5	7.2	32.4	8.9	32.3	8.7	32.5	7.7	31.7	6.5	0.67	0.611	0.002	n.s.

*M* mean, *SD* standard deviation, *STAI-T* State-Trait Anxiety Inventory – Trait, *ASI-3* Anxiety Sensitivity Index 3, *ACQ* Agoraphobic Cognition Questionnaire, *SPAI* Social Phobia and Anxiety Inventory, *LSAS* Liebowitz Social Anxiety Scale, *CES-D* Center for Epidemiological Studies-Depression Scale, *BIS* Behavioral Inhibition Scale, *GSE* General Self-Efficacy Scale, *CTQ* Childhood Trauma Questionnaire

^a^Clusters with the same letters do not differ on a Scheffé-corrected alpha level of 0.05. Alphabetical order (a to e) indicates cluster ranking (high to low) on each variable

In sum, these additional analyses suggest that cluster 5 is the most relevant for fear pathology as its members exhibit the highest psychometric characteristics of fear. Importantly, however, the defining features of this cluster during generalization, i.e., strongest mean fear responses and low CS-differentiation, were already apparent during fear acquisition.

## Discussion

Increased generalization of fear is considered crucial for the development of pathological fear and anxiety^8^. Following the Research Domain Criteria (RDoC) approach^21,22^ we expected that characteristic patterns of fear generalization also exist in healthy individuals and that some patterns may constitute a risk profile. Based on a cluster analysis to identify individual variations of fear generalization in a large sample of healthy participants examined with a generalization test following a differential fear conditioning protocol, we evaluated the stability and validity of the identified patterns. The analyses revealed five distinctive subgroups, respectively phenotypes of fear generalization. Relatively large subgroup sizes (*N* between 117 and 331) suggest that the clusters cover variance of naturally occurring individual differences and do not represent small extreme groups or outliers.

The clusters’ stability and characteristics were examined by means of three measures delineating the generalization gradients, i.e., mean response level, CS-differentiation, and linear deviation score (LDS). It is important to mention, that these dimensions reflect related processes in the context of fear generalization. Heightened mean response levels may in part reflect overgeneralization from CS+ to CS− and may be equally associated with a reduced CS-differentiation. On the other hand, CS-differentiation assesses the extent to which participants discriminate between two stimuli and thus might be inversely related to fear overgeneralization. Although correlational analyses confirmed these theoretical assumptions, mean response levels do not always map on CS-differentiation or linear deviation scores and vice versa. Therefore, we exploited the benefits of cluster analysis based on individual fear generalization gradients to delineate systematic co-variance between mean response levels, CS-differentiation and LDS. The stability of the generalization patterns identified on the basis of arousal ratings could be replicated reliably in randomly split subsamples and also for valence and US-expectancy ratings, as well as to some extent for skin conductance responses. We conclude that the observed clusters are stable^47^ and that the identified patterns are consistent over different dimensions of fear responses^5^, although the descriptive SCR generalization effects lack statistical significance and have an extremely small effect size ($\eta _p^2$ = 0.009). Nevertheless, we infer that generalization effects may vary based on the used measure of fear. Until now, overgeneralization effects related to anxiety disorders were mostly revealed using startle responses^14,15^ which were not recorded in the current study.

The analyses of the clusters’ characteristics suggest that cluster 5 is the most relevant for fear pathology since it shows strongest mean fear responses, low CS-differentiation, and strongest fear generalization. Indeed, previous studies revealed that strong mean fear responses^50^, reduced CS-differentiation^51^ and/or increased fear generalization^14,15^ are related to anxiety disorders. Clusters 3 and 4 show some markers of fear psychopathology too as both are characterized by relatively strong mean fear responses plus either low CS-differentiation or strong fear generalization, respectively. In contrast, clusters 1 and 2 seem to represent low risk clusters as they are characterized by low fear responses and low fear generalization, and cluster 2 in addition by good CS-differentiation. We conclude that the identified clusters vary in the degree of shared characteristics of fear generalization with anxiety disorder patients. Especially cluster 5 seems to share characteristics with patient samples and may thus represent a risk group for fear pathology.

Further analyses suggest three additional important conclusions. First, the patterns of mean fear responses during conditioning and generalization were mirrored by most psychometric measures of fear, with highest psychometric characteristics of fear in members of cluster 5 compared with cluster 1. Second, neither CS-differentiation nor GS-linearity but mean responses—averaged across all stimuli—explained most individual variance between the different subgroups. Third, the patterns of mean responses, especially the strong mean responses of cluster 5 as compared with cluster 1, were already apparent during fear acquisition. These findings have important implications for previous and future research on fear generalization and conditioning.

First, and most importantly, earlier studies in this domain emphasized the importance of increased generalization as a crucial characteristic of anxiety disorder patients^14,15^, however, these findings have rarely been related to differences in mean fear responses. Therefore, we systematically evaluated these studies on fear generalization in anxiety disorder patients regarding mean fear response levels, and consistently found differences corroborating our observation. For example, GAD patients compared with healthy control participants were found to overgeneralize conditioned fear in terms of more linear generalization gradients, but they also exhibited increased risk ratings for all stimuli (see Fig. 3 in Lissek et al.^14^) reflecting a general difference in the mean responding to fear stimuli between patients and controls. The same holds true for reports on overgeneralization of fear in patients with panic disorder compared with healthy controls, with patients also characterized by generally increased fear responses^15^. Furthermore, patients with social anxiety disorder did not display overgeneralization of conditioned fear, although they demonstrated increased mean fear responses compared with healthy controls^17^. We conclude that the cluster 5 phenotype revealed here in healthy individuals is the most similar to patients with anxiety disorders regarding both the increased linearity of generalization gradients and the generally increased fear responses and thus might constitute the most valid intermediate phenotype of anxiety disorders. In contrast, the cluster 1 phenotype, characterized by a non-linear gradient with low mean fear responses is virtually the most opposite to patients with anxiety disorders, with the remaining clusters varying in their characteristics between these two extremes. This interpretation is in line with the RDoC framework’s idea that mental disorders reflect extreme phenotypes on a dimensional continuum^22,52^. Thus, the revealed clusters, and especially clusters 1 and 5 as the two most extreme phenotypes, may help to identify risk and protective factors for anxiety disorders. Importantly, based on our findings we speculate that the mean fear response levels during conditioning and generalization are most important for characterizing the dimensional risk.

Second, our interpretation is in line with recent meta-analyses^50,51^ concluding that anxiety patients are characterized by elevated fear responses especially to safety cues. Our results support this notion as our risk cluster 5 showed strong fear responses to both the threatening cue as well as to the safety cue already during acquisition, and this response pattern during acquisition was the best predictor of later fear generalization. Thus, generally enhanced fear responses may give rise to stimulus overgeneralization.

Third, our interpretation is supported by the analyzed questionnaire data as the differences between clusters in mean fear responses were grossly mirrored by differences in anxiety-related personality traits. Participants in the supposedly critical cluster 5 had higher scores in psychometric measures of general and social anxiety than cluster 1. However, no psychometric measure allowed for validly differentiating between the other clusters (clusters 2, 3, and 4), suggesting that anxiety-related personality traits differentiate only between the most extreme phenotypes and that the experimentally assessed mean fear responses during conditioning and generalization are more sensitive to reveal phenotypical characteristics. This interpretation is in line with previous findings that failed to discern any effect of trait-anxiety on fear generalization^53,54^. We conclude that experimentally assessed behavioral phenotypes vs. psychometrically assessed symptoms of anxiety correlate only for the most extreme phenotypes.

This study has important strengths, e.g., large sample size, stringent methodological approach and analysis of generalization patterns on multiple levels, but also some weaknesses. First, we had no a priori hypothesis about the number of clusters. Even though the cluster patterns could be retrieved in several split-half samples, future confirmatory analyses should be conducted with different generalization paradigms (e.g., by using other dimensions for constructing conditioned and generalization stimuli) to confirm the reliability and validity of the identified phenotypes. Second, future longitudinal studies should examine the predictive value of the identified phenotypes, and studies including anxiety disorder patients too should clarify whether patients can be allocated to the most extreme cluster 5 or form an entirely separate group. Moreover, the majority of the present sample consists of relatively young, well-educated individuals with a Caucasian descent (up to the third generation), which restricts generalization of the present findings to a broader population. Finally, the clusters revealed here should be probed as intermediate phenotypes of anxiety disorders in neurobiological, e.g., genetic/epigenetic studies^12^.

In conclusion, the present study is the first to systematically investigate individual variance of fear generalization in a large sample of healthy participants. By using cluster analysis on individual fear generalization gradients, we reliably identified five different subtypes of behavioral fear responding. The clusters’ characteristics seem to reflect a dimensional risk factor varying in mean fear response and strength of generalization. The most extreme subgroups likely constitute a risk cluster and a resilient cluster, respectively, characterized by high vs. low mean fear response and strong vs. weak fear generalization plus high vs. low levels of anxiety traits. However, regarding the well-established link between anxiety traits and the risk for anxiety disorders^55^, it seems that the behavioral phenotype and the psychometrically assessed phenotype are relatively independent at least in healthy individuals and should be considered as two separable risk factors for the development of pathological forms of fear and anxiety. Our results further emphasize that the group characteristics explaining most variance, i.e., increased mean fear responses and reduced CS-differentiation, are already evident during fear conditioning, a finding challenging the assumed unique importance of overgeneralization as a crucial risk factor for anxiety disorders. However, we acknowledge that because of the combination of high mean response levels and overgeneralization in Cluster 5, we cannot definitely infer which dimension contributes most to high levels of anxiety-related traits.

## Supplementary information

Supplemental Material

Supplemental Figure S1

Supplemental Figure S2

Supplemental Figure S3

Supplemental Figure S4
