# Supplementary material for: Individual differences in human fear generalization—pattern identification and implications for anxiety disorders
Source: Transl Psychiatry. 2019 Nov 18;9:307. doi: 10.1038/s41398-019-0646-8 (PMC6861247; doi:10.1038/s41398-019-0646-8)
Supplement: Supplementary file 1 — Supplemental Material [file 41398_2019_646_MOESM1_ESM.docx]

**Supplemental Material for:**

**Individual Differences in Human Fear Generalization – Pattern Identification and Implications for Anxiety Disorders**

Authors: Y. Stegmann, MSc^1,2,#^, M. A. Schiele, PhD^3,#^, D. Schümann, PhD^4^, T. B. Lonsdorf, PhD^4^, P. Zwanzger, MD^5,6,7^, M. Romanos, MD^8^, A. Reif, MD^2,9^, K. Domschke, MA, MD, PhD^2,3,10^, J. Deckert, MD^11^, M. Gamer, PhD^1,2,§^, P. Pauli, PhD^1,2,§,*^

^1^ Department of Psychology, University of Würzburg, Würzburg, Germany

^2^ Center for Mental Health, University of Würzburg, Germany

^3^ Department of Psychiatry and Psychotherapy, Medical Center - University of Freiburg, Faculty of Medicine, University of Freiburg, Freiburg, Germany

^4^ Department of Systems Neuroscience, University Medical Center Hamburg-Eppendorf, Hamburg, Germany

^5^ kbo-Inn-Salzach-Hospital, Wasserburg, Germany

^6^ Department of Psychiatry, University of Münster, Germany

^7^ Department of Psychiatry, Ludwig-Maximilian-University of Munich, Germany

^8^ Department of Child and Adolescent Psychiatry, Psychosomatics and Psychotherapy, Center of Mental Health, University Hospital Würzburg, Germany

^9^ Department of Psychiatry, Psychosomatic Medicine and Psychotherapy, University Hospital Frankfurt, Frankfurt am Main, Germany

^10^ Center for NeuroModulation, Faculty of Medicine, University of Freiburg, Germany

^11^ Department of Psychiatry, Psychosomatics and Psychotherapy, Center of Mental Health, University Hospital Würzburg, Germany

^#^These authors share first authorship.

^§^These authors share senior authorship.

*Corresponding Author

Prof. Dr. Paul Pauli

Department of Psychology (Biological Psychology, Clinical Psychology, and Psychotherapy), and Center of Mental Health, University of Würzburg, Marcusstraße 9-11, 97070 Germany

E-Mail: [Pauli@mail.uni-wuerzburg.de](mailto:Pauli@mail.uni-wuerzburg.de)

**Supplemental Figures and Tables**

**Supplemental Figure S1**: (Left) Within cluster sum of squares screeplot for a two to fifteen cluster solution using a k-means clustering algorithm. The optimal number of clusters is identified by the “bend” in the plot, which indicates an inconsistent increase in the within cluster sum of squares (elbow-criterion). Visual inspection of the scree plot suggests a four, five or six cluster solution. (Right) Frequencies of the proposed numbers of clusters as determined by 25 different objective indices (1).

**Supplemental Figure S2:** Arousal generalization gradients for each cluster, after dividing the whole sample into two halves and repeating the cluster analysis procedure on both subsamples. The resulting cluster structure and sample sizes were similar to the whole sample cluster analysis, suggesting a strong stability of the five cluster solution. Error bars represent standard errors of the mean.

**Supplemental Figure S3:** Valence generalization gradients for each cluster (A) and corresponding gradient parameters (B-D). For better interpretability, the valence-axis was reversed (negative valence up). Mean valence responses increased from cluster 1 to 5 (B); CS-differentiation was better in clusters 2 and 4 than clusters 1, 3 and 5 (C); LDS was lower in clusters 4 and 5 than clusters 1, 2 and 3 (D). Clusters with the same letters do not differ on a Scheffé-corrected alpha level of .05. Error bars represent standard errors of the mean.

**Supplemental Figure S4:** US-Contingency generalization gradients for each cluster (A) and corresponding gradient parameters (B-D). Mean contingency responses increased from cluster 1 to 5 (B); CS-differentiation was better in cluster 2 and 4 than clusters 1, 3 and 5 (C); LDS was lower in clusters 4 and 5 than clusters 1,2 and 3 (D). Clusters with the same letters do not differ on a Scheffé-corrected alpha level of .05. Error bars represent standard errors of the mean.

**Supplemental Table 1:** Cluster comparisons on generalization gradient parameters.

|  | Cluster 1 | |  | Cluster 2 | |  | Cluster 3 | |  | Cluster 4 | |  | Cluster 5 | |  | Statistics |  |  | Cluster: |
| --- | --- | --- | --- | --- | --- | --- | --- | --- | --- | --- | --- | --- | --- | --- | --- | --- | --- | --- | --- |
|  | (n = 240) | |  | (n = 331) | |  | (n = 251) | |  | (n = 236) | |  | (n = 117) | |  |  |  |  | 1 - 2 - 3 - 4 - 5 |
|  | M | SD |  | M | SD |  | M | SD |  | M | SD |  | M | SD |  | *F*(4,1170) | *p* | $\eta_{p}^{2}$ | Scheffé^1^ |
| Arousal: |  |  |  |  |  |  |  |  |  |  |  |  |  |  |  |  |  |  |  |
| Level | 2.06 | .52 |  | 3.29 | .45 |  | 4.24 | .51 |  | 4.62 | .52 |  | 5.98 | .70 |  | 1450.50 | < .001 | .832 | e - d - c - b - a |
| CS-Diff. | 1.71 | 1.60 |  | 4.84 | 1.42 |  | 1.35 | 1.70 |  | 5.24 | 1.34 |  | 2.21 | 1.86 |  | 348.06 | < .001 | .543 | bc - a - c - a - b |
| LDS | .80 | .86 |  | 1.42 | 0.87 |  | .66 | 1.12 |  | -.04 | .95 |  | .07 | .95 |  | 96.84 | < .001 | .249 | b - a - b - c - c |
| Valence: |  |  |  |  |  |  |  |  |  |  |  |  |  |  |  |  |  |  |  |
| Level | 3.87 | 1.22 |  | 4.02 | .80 |  | 4.76 | .68 |  | 4.82 | .62 |  | 5.80 | .93 |  | 136.99 | < .001 | .319 | c - c - b - b - a |
| CS-Diff. | 1.20 | 1.59 |  | 3.84 | 2.13 |  | 1.39 | 1.82 |  | 4.63 | 2.05 |  | 2.06 | 2.18 |  | 152.16 | < .001 | .342 | d - b - cd - a - c |
| LDS | .76 | .91 |  | 1.32 | 1.04 |  | .74 | 1.06 |  | .27 | .96 |  | .14 | .92 |  | 52.32 | < .001 | .152 | b - a - b - c - c |
| Contingency: |  |  |  |  |  |  |  |  |  |  |  |  |  |  |  |  |  |  |  |
| Level | 22.4 | 14.9 |  | 25.4 | 10.9 |  | 32.3 | 14.3 |  | 32.9 | 13.7 |  | 41.4 | 18.2 |  | 49.88 | < .001 | .146 | c - c - b - b - a |
| CS-Diff. | 49.9 | 26.4 |  | 67.6 | 21.0 |  | 53.0 | 26.9 |  | 70.9 | 21.1 |  | 52.2 | 29.8 |  | 37.88 | < .001 | .115 | b - a - b - a - b |
| LDS | 18.4 | 13.9 |  | 22.0 | 12.6 |  | 17.7 | 14.1 |  | 14.4 | 12.4 |  | 10.1 | 13.6 |  | 22.09 | < .001 | .070 | b - a - bc - cd - d |
| SCR: |  |  |  |  |  |  |  |  |  |  |  |  |  |  |  |  |  |  |  |
| Mean | .08 | .06 |  | .10 | .09 |  | .10 | .08 |  | .10 | .09 |  | .13 | .11 |  | 8.12 | < .001 | .027 | c - b - bc - ab - a |
| CS-Diff. | .02 | .09 |  | .05 | .11 |  | .04 | .10 |  | .07 | .14 |  | .05 | .13 |  | 5.86 | < .001 | .019 | b - ab - ab - a - ab |
| LDS | .00 | .04 |  | .02 | .05 |  | .01 | .06 |  | .01 | .06 |  | .02 | .05 |  | 2.64 | .032 | .009 | n.s. |

^1^ Clusters with the same letters do not differ on a Scheffé-corrected alpha level of .05. Alphabetical order (a to e) indicates cluster ranking (high to low) on each variable.

M = mean, SD = standard deviation, Diff. = differentiation between CS+ and CS-, LDS = linear deviation score, SCR = skin conductance responses.

**Supplemental Table 2:** Cluster comparisons on acquisition data.

|  | Cluster 1 | |  | Cluster 2 | |  | Cluster 3 | |  | Cluster 4 | |  | Cluster 5 | |  | Statistics |  |  | Cluster: |
| --- | --- | --- | --- | --- | --- | --- | --- | --- | --- | --- | --- | --- | --- | --- | --- | --- | --- | --- | --- |
|  | (n = 240) | |  | (n = 331) | |  | (n = 251) | |  | (n = 236) | |  | (n = 117) | |  |  |  |  | 1 - 2 - 3 - 4 - 5 |
|  | M | SD |  | M | SD |  | M | SD |  | M | SD |  | M | SD |  | *F*(4,1170) | *p* | $\eta_{p}^{2}$ | Scheffé^1^ |
| Arousal: |  |  |  |  |  |  |  |  |  |  |  |  |  |  |  |  |  |  |  |
| Level | 2.86 | 1.12 |  | 4.29 | 1.06 |  | 4.69 | 1.02 |  | 4.63 | 1.01 |  | 5.83 | 1.14 |  | 185.02 | < .001 | .387 | d - c - b - b - a |
| CS-Diff. | 1.68 | 2.05 |  | 3.89 | 2.22 |  | 1.52 | 2.26 |  | 4.46 | 1.87 |  | 1.81 | 2.25 |  | 102.76 | < .001 | .260 | c - b - c - a - c |
| Valence: |  |  |  |  |  |  |  |  |  |  |  |  |  |  |  |  |  |  |  |
| Level | 4.44 | 1.33 |  | 4.88 | 1.05 |  | 5.09 | .97 |  | 5.01 | .93 |  | 5.76 | 1.14 |  | 31.13 | < .001 | .096 | c - b - b - b - a |
| CS-Diff. | 1.25 | 1.88 |  | 3.16 | 2.47 |  | 1.18 | 2.21 |  | 4.06 | 2.16 |  | 1.72 | 2.45 |  | 84.69 | < .001 | .225 | c - b - c - a - c |
| Contingency: |  |  |  |  |  |  |  |  |  |  |  |  |  |  |  |  |  |  |  |
| Level | 47.6 | 12.7 |  | 49.4 | 10.9 |  | 52.5 | 13.5 |  | 50.0 | 9.3 |  | 55.6 | 16.5 |  | 10.98 | < .001 | .036 | c - bc - bc - ab - a |
| CS-Diff. | 55.4 | 37.2 |  | 68.1 | 29.9 |  | 48.5 | 34.5 |  | 71.0 | 24.5 |  | 49.6 | 33.1 |  | 24.89 | < .001 | .078 | b - a - b - a - b |
| SCR: |  |  |  |  |  |  |  |  |  |  |  |  |  |  |  |  |  |  |  |
| Mean | .09 | .09 |  | .12 | .11 |  | .13 | .11 |  | .12 | .12 |  | .16 | .13 |  | 8.33 | < .001 | .029 | c - bc - b - ab - a |
| CS-Diff. | .01 | .10 |  | .03 | .12 |  | .00 | .12 |  | .02 | .11 |  | .02 | .11 |  | 3.57 | .007 | .012 | ab - a - b - ab - ab |

^1^ Clusters with the same letters do not differ on a Scheffé-corrected alpha level of .05. Alphabetical order (a to e) indicates cluster ranking (high to low) on each variable.

M = mean, SD = standard deviation, Diff. = differentiation between CS+ and CS-, SCR = skin conductance responses.
