# Supplementary figures and images for: Individual differences in human fear generalization—pattern identification and implications for anxiety disorders
Source: Transl Psychiatry. 2019 Nov 18;9:307. doi: 10.1038/s41398-019-0646-8 (PMC6861247; doi:10.1038/s41398-019-0646-8)

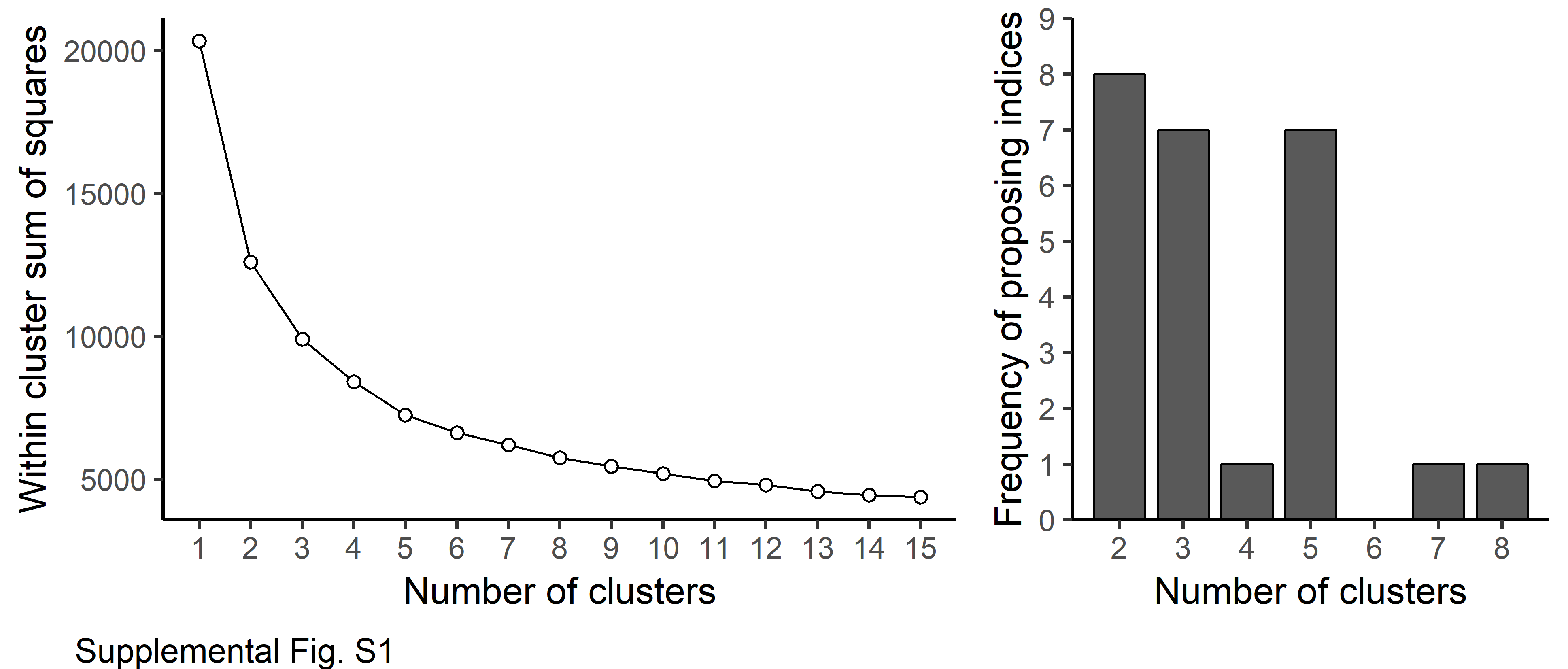

Supplement: Supplementary file 2 — Supplemental Figure S1 [file 41398_2019_646_MOESM2_ESM.tif]

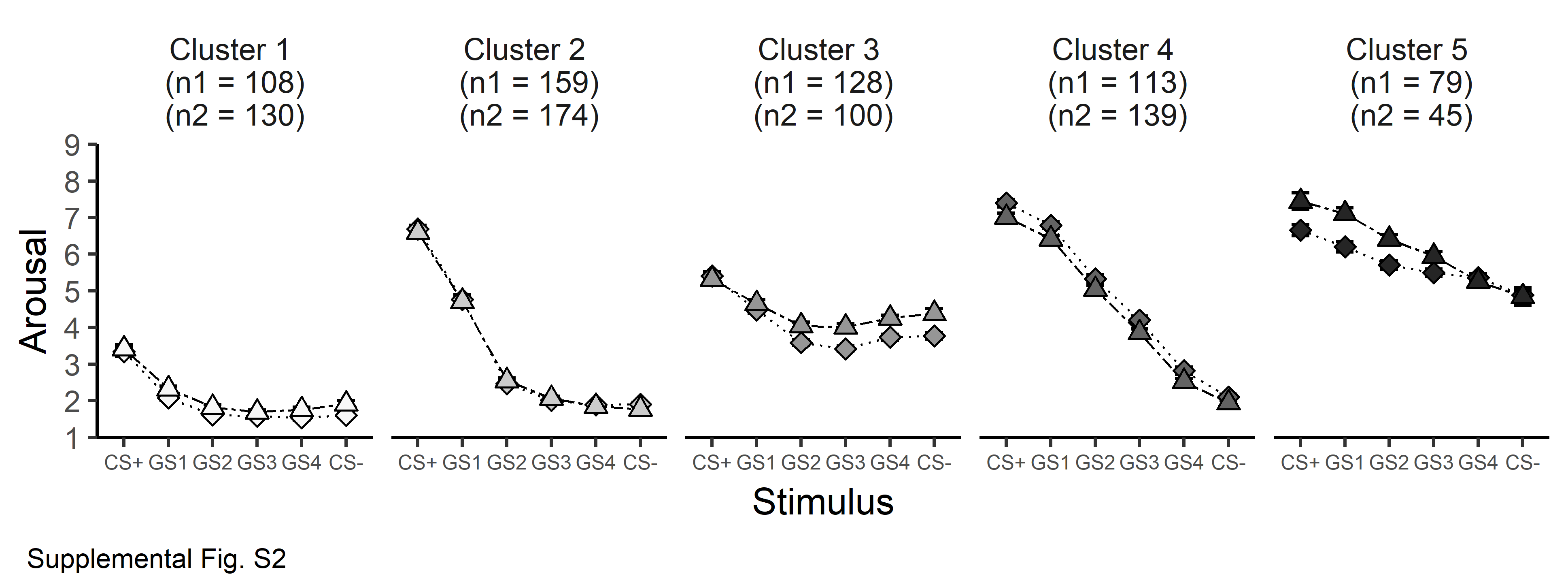

Supplement: Supplementary file 3 — Supplemental Figure S2 [file 41398_2019_646_MOESM3_ESM.tif]

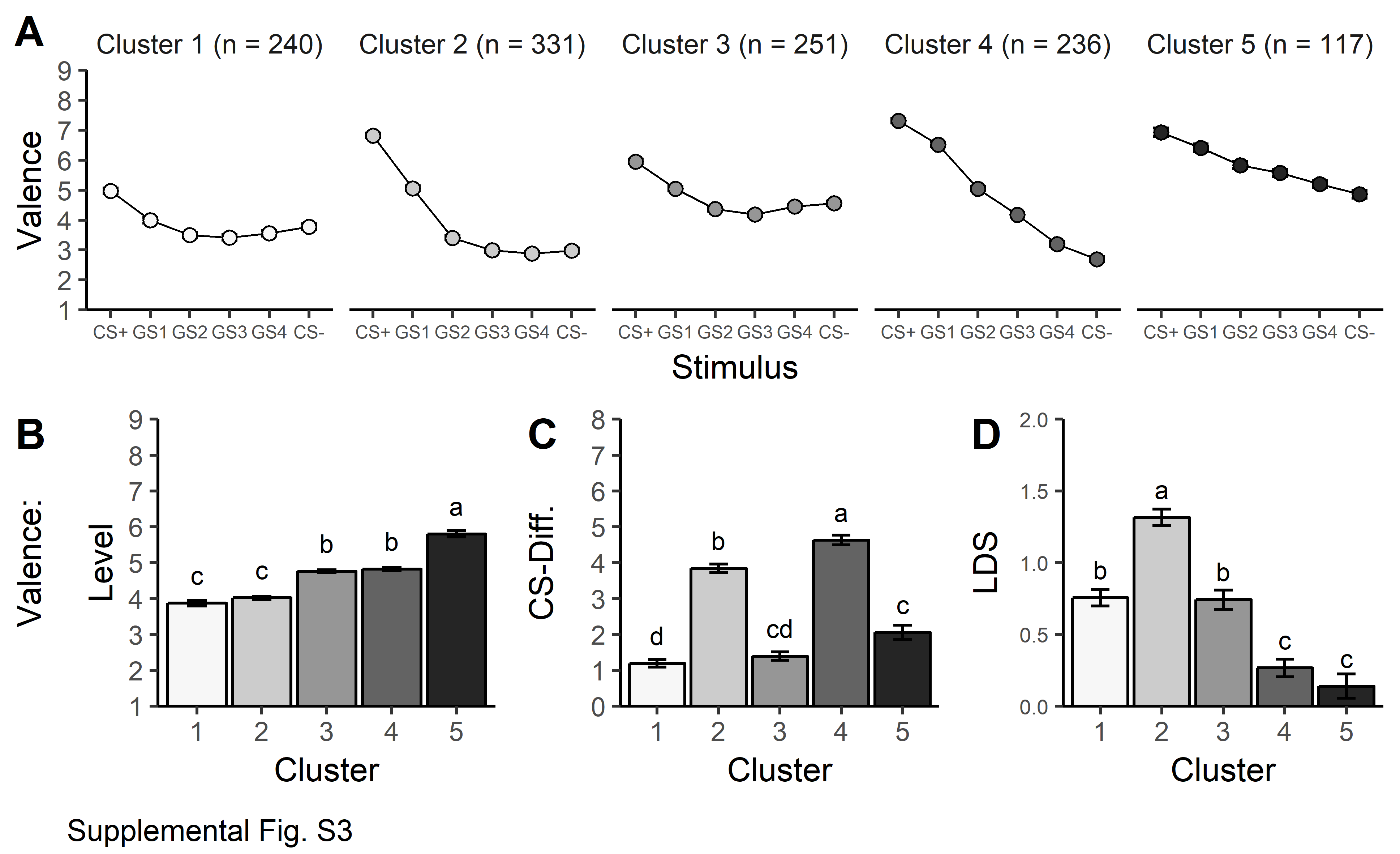

Supplement: Supplementary file 4 — Supplemental Figure S3 [file 41398_2019_646_MOESM4_ESM.tif]

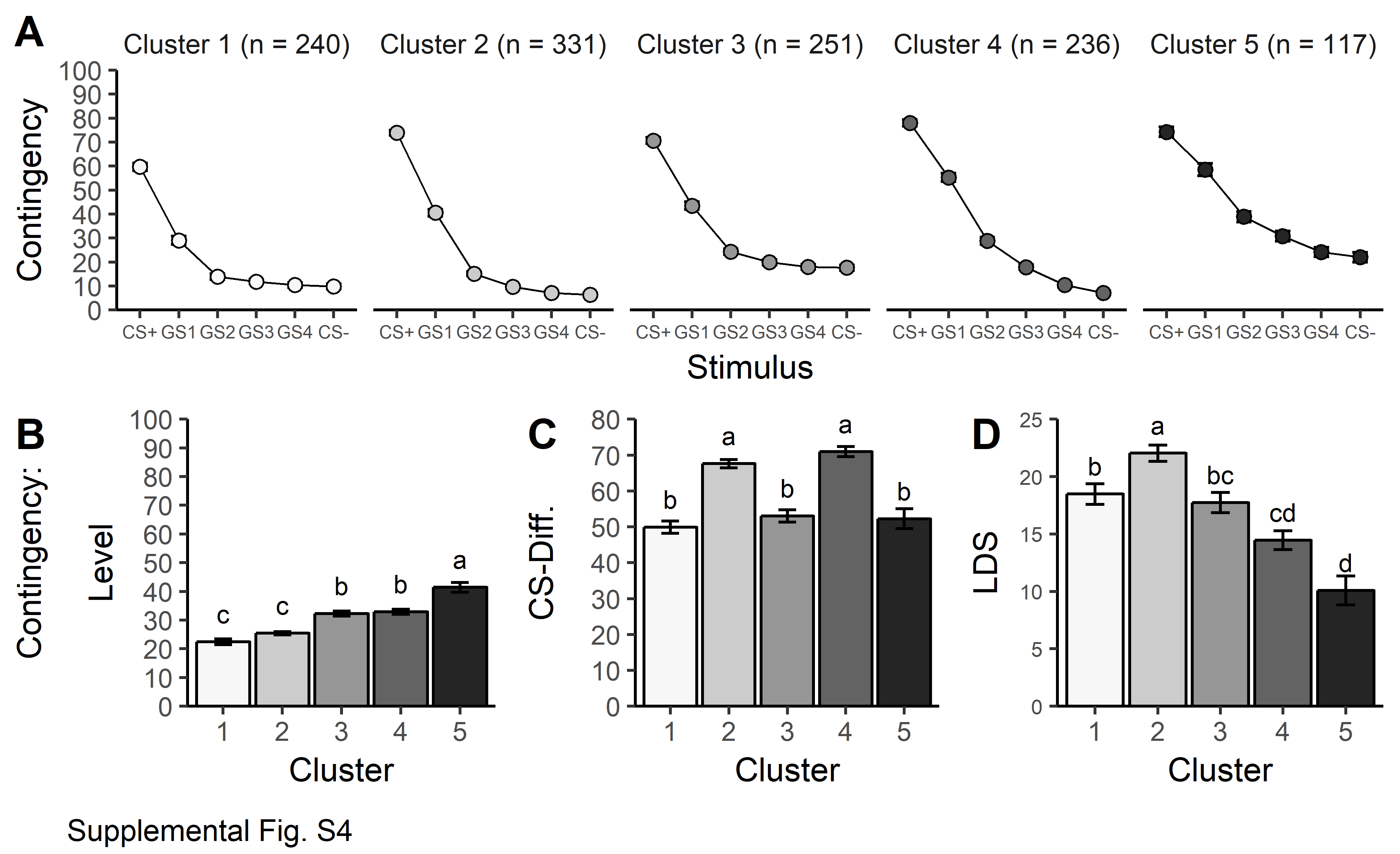

Supplement: Supplementary file 5 — Supplemental Figure S4 [file 41398_2019_646_MOESM5_ESM.tif]
